# The Medical Letter Bag

**Published:** 1919-03

**Authors:** 


### "The Medical Letter-Bag."

For some time The Texas Medical Journal has thought of setting aside a few pages for letters from physicians on subjects they may think of interest to the profession. There is nothing that brings people together quite so much as chatty little talks just among themselves on whatever may at the moment be uppermost in their minds. The Texas Medical Journal is anxious to become the medium for those little exchanges of opinion among doctors. In that way it can render a nice service for its friends. By turning to that department today you will find an interesting suggestion from Dr. E. H. Martin, of Hot Springs. Perhaps the letter will start you thinking along that line or some thought entirely foreign to that may occur to you. Pass your thoughts on to others. "Thoughts are valuable in proportion as they are generative." They are worth nothing until they are adequately placed.

It is hoped that this "Medical Letter-Bag" may be filled to overflowing with good suggestions. If you have had an experience or have had an observation that hardly justifies a lengthy article, sit down and tell it briefly to the "Letter-Bag." Little things occur every day in the practice of almost every doctor that hundreds of others would like to know about. Don't be selfish enough to keep these all to yourself but take the few minutes necessary to let others know about them.

